# Gloucester General Infirmary

**Published:** 1831-10

**Authors:** 


### GLOUCESTER GENERAL INFIRMARY.

### *Strangulated Scrotal Hernia, with an Encysted Hydrocele of the Chord in front of it taken for the Sac of the Hernia, with other anomalous Circumstances.**

After making his external incision very low on the tumor, the surgeon thought, after some dissection, that he had reached the sac, and accordingly opened it in a cautious manner, when his belief was confirmed by the escape of a considerable quantity of serum from the cavity. This was slit up extensively; its parietes were very thick.

The contents of this supposed sac appeared to be cœcum; for there lay, at the extremity of this last-named resemblance, a part, which looked like the appendix vermiformis.

Insuperable difficulties, however, at the neck of this supposed sac, interrupted the regular progress of the operation; the hernia could not be returned: the operator divided the tendon, and quitted his patient.

Some time afterwards, in consequence of the symptoms of strangulation remaining in all their force, another examination was made of the parts concerned in the operation.

It was discovered that the substance which appeared to be the vermiform appendix of the cœcum, was the testis, with its vessels,

* Fletcher's Med. Chir. Notes.

very much dwindled in size, and which lay at the extremity of the hernial sac in such a way as to make it appear like the cœcum itself.

The hernial sac, therefore, was yet to be opened, which was accordingly done; the tendon cut again; and the hernia, which was a portion of the ilium, returned. The patient recovered.

The perplexing and irregular circumstances of the foregoing case arose out of the novelty of an encysted hydrocele of the chord being found in front of the hernia, without the chord itself occupying that situation. The hernia, in descending through the loose cellular membrane between the chord and the cremaster muscle, had condensed some watery cysts against the latter, in such a manner that a complete encysted hydrocele was the result, containing about two tablespoonsful of a serous fluid; the escape of which had deceived the operator into a belief that he had opened the true hernial sac.

The cremaster muscle, with the condensed cellular texture, formed a very thick and strong front to this watery tumor, which was moulded thinly by an old hernia over its own front, so as not to disturb its smooth convex character. This latter circumstance, together with the immobility of the whole tumor, composed, as it was, of two distinct parts, which it was impossible to distinguish from each other, left the operator without any other guide than the knife to ascertain the real composition of the mass. When the knife had penetrated the watery cyst, and the parts were further opened, he was still further misled, and confirmed in his belief that he had entered the sac of the hernia, from seeing the dwindled testis and hernia lying together, in a condition to resemble the cœcum with its appendage.

The subsequent history of the case will, however, serve to shew the necessity of vigilance, of extreme care and deliberation in opening herniary tumors. To avoid confusion in making out the identity of parts, the natural ones ought first to be traced. Had the testis been sought for in the foregoing example, the inquiry must have led to its detection at the extremity of the protrusion, which then, in its turn, would have undergone a more rigorous examination, and its real character have been quickly developed.

---

